# Treatment of Congenital Absence of the Mammary Gland

**DOI:** 10.1155/2013/676573

**Published:** 2013-02-12

**Authors:** Masaki Yazawa, Mika Watanabe, Masahiro So, Kazuo Kishi

**Affiliations:** ^1^Plastic and Reconstructive Surgery, School of Medicine, Keio University, 35 Shinanomachi, Shinjuku-ku, Tokyo 160-8582, Japan; ^2^Keiyu Plastic Clinic, 3-5-7 Funabori, Edogawa-ku, Tokyo 134-0091, Japan

## Abstract

Breast reconstruction for breast deformity is significant not only for esthetic purposes but also from a psychological perspective. There have been a few reports on treatment of congenital simple absence of the mammary gland. For patients in puberty, even if they are in the middle of the growth phase, breast reconstruction is very important for the mental quality of life. In our two cases of congenital absence of unilateral mammary gland, breast reconstruction with a tissue expander worked well in terms of esthetic results and the psychological condition of the young patients. In our institute, operative indications are as follows: (1) a girl over 15 years old (this age is selected as breast growth can be determined at this time), (2) no endocrine-related disorders, (3) preoperative examination of breast MRI or US showing the absence or significant hypoplasia of mammary gland, and (4) a wish for breast reconstruction by the patient herself. For patients in the middle of the growth phase, silicone breast implant does not require a donor site and is easily adjustable in terms of volume to match the growth of the breast on the unaffected side by exchanging the silicone breast implant. Therefore, silicone breast implant is a better procedure than skin flaps with their accompanying large donor sites.

## 1. Introduction

Breast reconstruction for breast deformity is significant not only for esthetic purposes but also from a psychological perspective. Various procedures have been reported for various conditions or causes of breast deformity and asymmetry. Among congenital breast deformity diseases that require surgical treatment at puberty, Poland's syndrome is one of the most famous [1–3]. However, it is accompanied by thorax deformity and the absence of pectoralis major and is often reported to require a major operation with skin flaps. There have been a few reports on treatment of congenital simple absence of the mammary gland [4–7].

## 2. Patients and Methods

First, to rule out an unknown disease, the hormone contents of blood are measured and the presence of menstruation is checked. Symmetry of thorax and pectoralis major is examined by magnetic resonance imaging (MRI) or computer tomography (CT), and the condition of the mammary gland is checked by ultrasonography (US). Before breast implant insertion, a tissue expander is inserted into the submuscular layer of the affected side of the pectoralis major. The tissue expander aims to compensate for the lack of skin at the craniad area of the nipple. Expansion is carried out at an outpatient clinic to determine an adequate size for the breast implant. Finally, the tissue expander is exchanged for a breast implant.

### 2.1. Case 1

**Figure 1:**
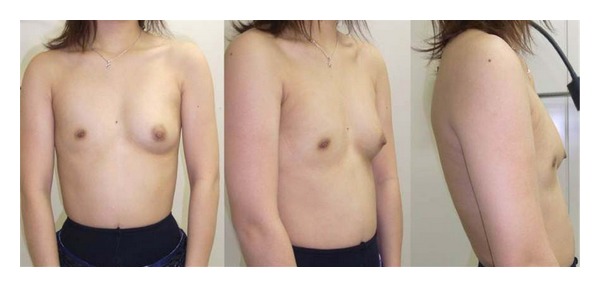
Preoperative view.

**Figure 2:**
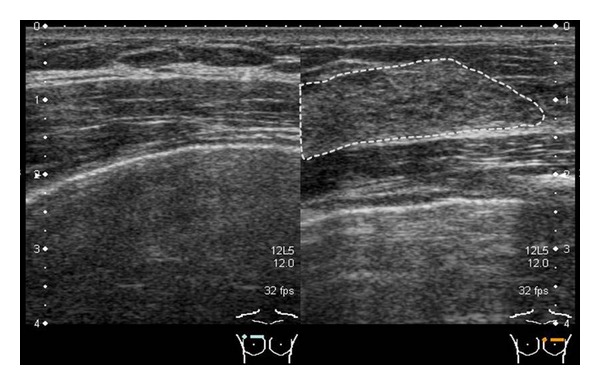
Ultrasound: thorax and pectoralis major are symmetric. Area framed by a broken line is a normal mammary gland on the unaffected side.

**Figure 3:**
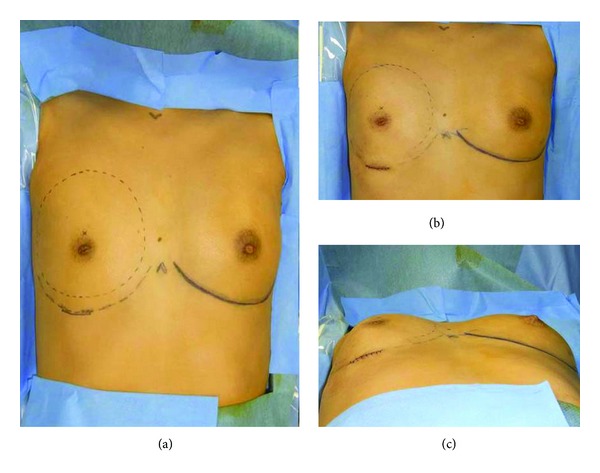
Preoperative view: (a) The quantity of skin was insufficient in the area above the nipple on the affected side. The center of the inserted tissue expander was positioned 2 cm above the nipple. Postoperative view: (b) Incision was 4 cm long and positioned at the expected inframammary fold after reconstruction. A tissue expander was inserted into the submuscular layer. Initial volume of the tissue expander was 50 mL (c).

**Figure 4:**
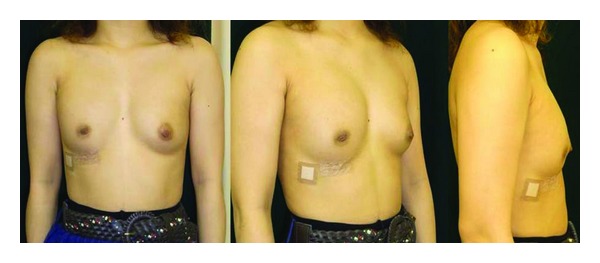
After expansion: the final volume of the tissue expander was 150 mL.

**Figure 5:**
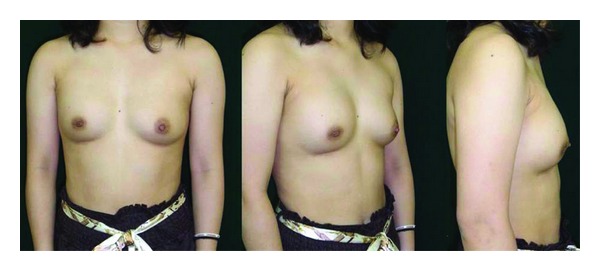
One year after operation, the positions of nipples and inframammary folds are symmetric.

Breast asymmetry was identified at the age of 15. The Tanner stages were B-2 on the left healthy side and B-1 on the right affected side (Figure 1). The hormone contents of blood were 8.90 for LH, 4.20 for FSH, and 82.2 for estradiol, which were within normal limits. The patient was a menstruating girl. In MRI and CT examinations, the thorax and pectoralis major were found to be symmetric, but the mammary gland tissue was absent on the right affected side and present on the left (Figure 2). The diameters of nipples were 12 mm on the right and 14 mm on the left, almost symmetric on both sides. Until the age of 18, the patient did not take part in swimming classes at her high school. Before college admission, the breast asymmetry became significant, and the patient decided to have surgical treatment for congenital absence of the right mammary gland. At first, a tissue expander (PMT round type, 12.5 cm in diameter, capacity 600 mL) was inserted into the submuscular layer of the right pectoralis major from the expected inframammary line incision (Figure 3). Finally, six months later, the tissue expander was exchanged for a mammary silicone implant (Mentor CPG 321 anatomical type, 135 mL in volume) (Figures 4 and 5). 

### 2.2. Case 2

**Figure 6:**
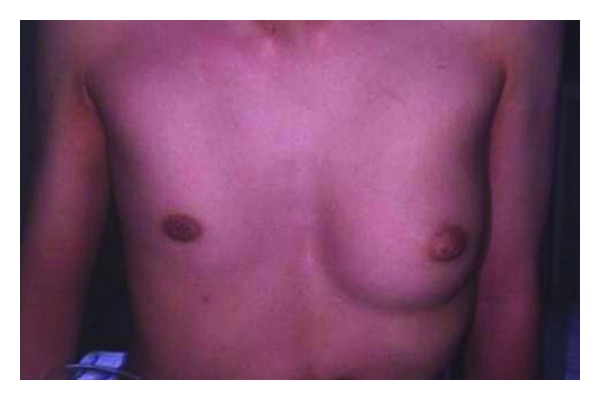
Preoperative view.

**Figure 7:**
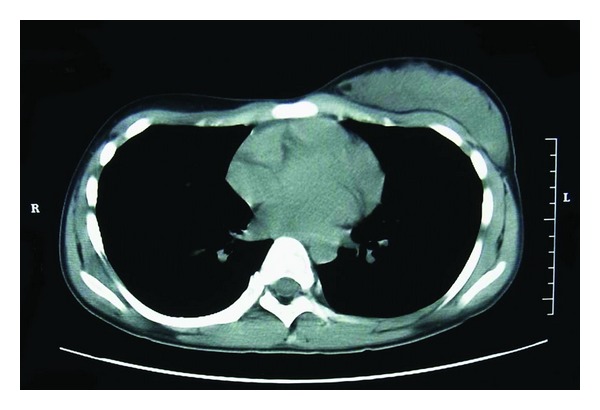
MRI: thorax and pectoralis major are symmetric. Normal mammary gland is present on the unaffected side.

**Figure 8:**
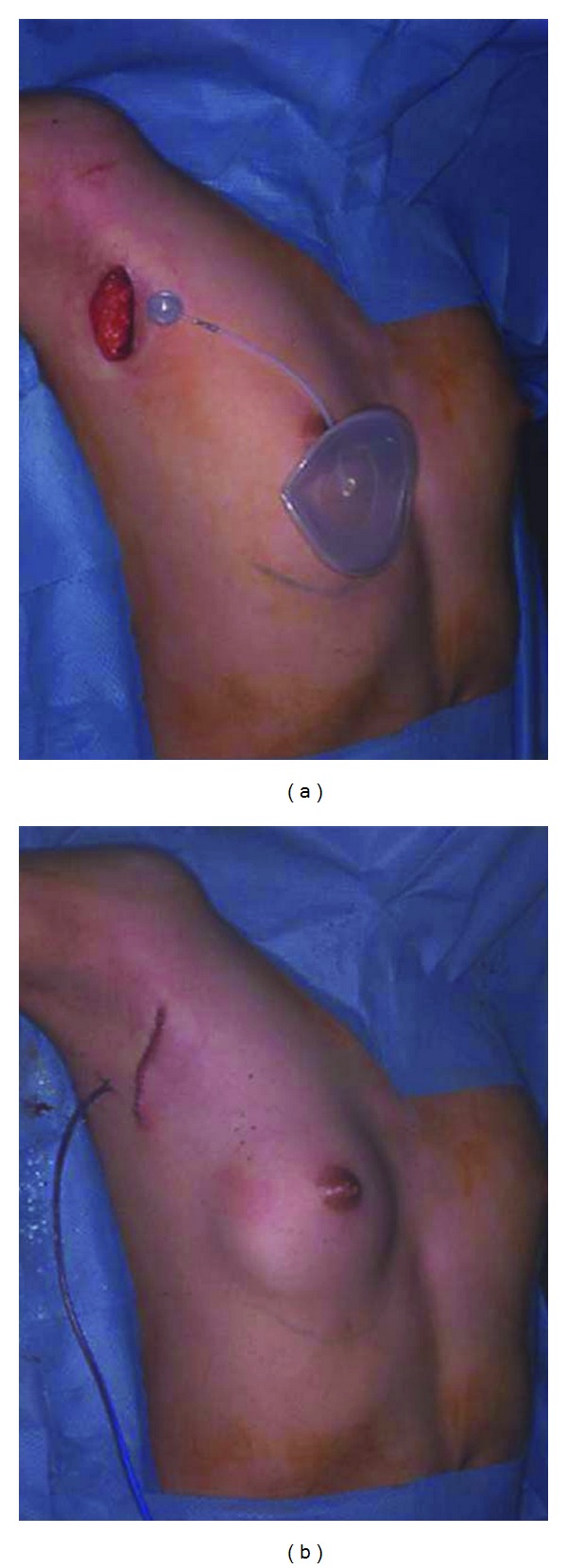
Intraoperative view: (a) Tissue expander was inserted submuscularly from the transaxial incision. (b) Initial volume of the tissue expander was 60 mL.

**Figure 9:**
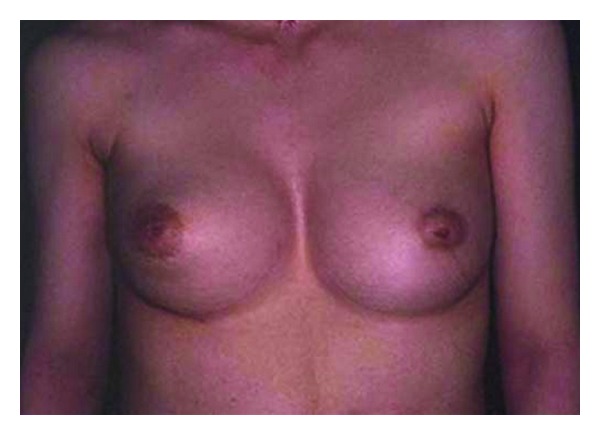
A saline-filled mammary prosthesis was inserted from the inframammary incision to achieve symmetry. Six months after the operation, the positions of nipples and inframammary folds are symmetric.

Breast asymmetry was identified at the age of 17. The patient had an amniocele operation, bilateral vocal cord paralysis, and scoliosis in her past history. The Tanner stages were B-4 on the left healthy side and B-1 on the right affected side (Figure 6). The patient was a menstruating girl. In CT examination, the thorax and pectoralis major were symmetric, but no development was seen on the right affected side of the mammary area (Figure 7). The breast asymmetry was significant, and the patient decided to have breast reconstruction. At first, a tissue expander (Koken round type, 15 cm in diameter, capacity 480 mL) was inserted into the submuscular layer of the right pectoralis major via a transaxial approach (Figure 8). Finally, six months later, the tissue expander was exchanged for a mammary silicone implant (Mentor Saline-Filled Mammary Prosthesis, 325 mL in volume) (Figure 9). 

## 3. Results

These two cases had no complications. The results were acceptable in terms of not only the esthetic outcome but also from a psychological perspective.

## 4. Discussion

One-sided breast aplasia or hypoplasia at the time of puberty can be a source of mental stress for children and may cause mental trauma. If a child diagnosed with the absence of mammary gland has no abnormal data at the time of endocrine examination, breast reconstruction should be indicated because the mammary gland would not be expected to develop in the future.

In autologous tissue transfer, for example latissimus dorsi musculocutaneous flap, rectus abdominis musculocutaneous flap, or deep inferior epigastric perforator flap (DIEP flap) is available for breast reconstruction. However, the use of the rectus abdominis has a disadvantage for childbearing. In addition, a breast reconstructed with DIEP flap or other musculocutaneous flaps can initially obtain symmetry but cannot be adjusted to fit the changing volume with the normal growth in the breast on the healthy side. On the other hand, silicone breast implant, approved by the U.S. Food and Drug Administration (FDA), does not require a donor site and is easily adjustable in terms of volume to match the growth of the breast on the unaffected side by exchanging the silicone breast implant. Therefore, silicone breast implant is a better procedure for girls in the growth phase. 

 In our institute, operative indications are as follows: (1) a girl over 15 years old (this age is selected as breast growth can be determined at this time), (2) no endocrine-related disorders, (3) preoperative examination of breast MRI or US showing the absence or significant hypoplasia of mammary gland, and (4) a wish for breast reconstruction by the patient herself. In our two cases of congenital absence of unilateral mammary gland, breast reconstruction with a tissue expander worked well in terms of esthetic results and the psychological condition of the young patients. 

## 5. Conclusion

Breast reconstruction for breast deformity is significant not only for esthetic purposes but also from a psychological perspective. For patients in puberty, even if they are in the middle of the growth phase, breast reconstruction is very important for the mental quality of life. For patients in the middle of the growth phase, silicone breast implant does not require a donor site and is easily adjustable in terms of volume to match the growth of the breast on the unaffected side by exchanging the silicone breast implant. Therefore, silicone breast implant is a better procedure than skin flaps with their accompanying large donor sites.
